# Disseminated Neocosmospora vasinfecta infection in a patient with acute nonlymphocytic leukemia.

**DOI:** 10.3201/eid0701.700149

**Published:** 2001

**Authors:** O A Cornely, J Chemnitz, H G Brochhagen, K Lemmer, H Schütt, D Söhngen, P Staib, C Wickenhauser, V Diehl, K Tintelnot

**Affiliations:** Klinik I für Innere Medizin, Universitätsklinik Köln, 50924 Köln, Germany. oliver.cornely@uni-koeln.de

## Abstract

We report Neocosmospora vasinfecta infection following chemotherapy for acute nonlymphocytic leukemia. N. vasinfecta, a plant pathogen, was identified by culture and genetic sequencing. Susceptibility testing revealed in vitro resistance for common antifungals.


### Disseminated *Neocosmospora vasinfecta* Infection in a Patient with Acute Nonlymphocytic Leukemia

**Oliver A. Cornely,* Jens Chemnitz,* Hans-Georg Brochhagen, Karin Lemmer,† Heidi Schütt, Dietmar Söhngen,* Peter Staib,* Claudia Wickenhauser,* Volker Diehl,* and Kathrin Tintelnot†**
*Universitätsklinik Köln, Köln, Germany, and
†Robert Koch-Institute, Berlin, Germany

We report *Neocosmospora vasinfecta* infection following chemotherapy for acute nonlymphocytic leukemia. *N. vasinfecta*, a plant pathogen, was identified by culture and genetic sequencing. Susceptibility testing revealed in vitro resistance for common antifungals.

*Neocosmospora vasinfecta*, a common plant pathogen in tropical and subtropical areas (1), is little known as a pathogen in humans. We report a case of fatal systemic *N. vasinfecta* infection in an African patient with leukemia. This is the third reported case of a human infection with this organism and the first case in which the infection was disseminated.

### The Case Study

A 38-year-old man was admitted to the University Hospital of Cologne, Germany, with a 4-week history of fatigue, vertigo, and bone pain. He had been living in Germany for 15 years and had returned from a holiday in Nigeria 2 weeks before admission. Past medical history included hepatitis C and malaria.

A bone marrow aspirate showed a predominance of atypical promyelocytes, suggesting acute nonlymphocytic leukemia FAB M3. The patient was treated with high-dose all-trans-retinoic acid for 14 days, followed by a first induction chemotherapy regimen consisting of cytarabine, daunorubicine, and thioguanine. Prophylactic treatment with fluconazole (400 mg/d) and trimethoprim-sulfamethoxazole (960 mg/d) was started because severe granulocytopenia, defined as an absolute neutrophil count below 100/µL of up to 4 weeks' duration, was anticipated.

Address for correspondence: Oliver A. Cornely, Klinik I für Innere Medizin, Universitätsklinik Köln, 50924 Köln, Germany; fax:+49-221-478-3611; e-mail: oliver.cornely@uni-koeln.de.

Pain projecting to the lateral side of both feet developed on day 12 of chemotherapy, when the patient's absolute neutrophil count had fallen below 100/µL. His feet were swollen and hyperthermic. Arthritis and deep vein thrombosis were ruled out. Pain resolved under symptomatic treatment with acetaminophen. On day 16 of induction therapy, bone marrow examination yielded persistence of blast cells. Therefore, a second induction chemotherapy was administered, despite ongoing granulocytopenia. The regimen comprised high-dose cytarabine and mitoxantrone. On day 16 after the onset of granulocytopenia, fever developed. Results of a chest X-ray and a high-resolution computerized tomography scan were unremarkable. Broad-spectrum antibiotic treatment was started, but the fever persisted. Several chest X-rays were nondiagnostic; however, after 24 days of granulocytopenia, another computerized tomography scan revealed interstitial and patchy lung infiltrates. Because of persistent fever and newly developing lung infiltrates under broad-spectrum antibiotic therapy, invasive fungal infection was suspected, and conventional amphotericin B was administered (1 mg/kg/d). After 1 week of antifungal therapy, the patient reported bilateral pleuritic chest pain. Because the patient's condition was deteriorating, antifungal therapy was altered to 2 mg/kg/day liposomal amphotericin B, but pulmonary infiltrates persisted. Granulocytopenia resolved after 34 days, and a bone marrow aspirate showed complete remission of leukemia. On the same day, an abdominal ultrasound exam revealed hepatosplenomegaly and hypodensic lesions in the liver, suggesting possible hepatosplenic involvement of fungal disease. A lateral purulent ulcerous lesion of the right foot developed, accompanied by worsening pain and swelling. Swab cultures revealed a mold that was not initially identified. Subsequently, the same mold was isolated from sputum samples, transtracheal aspirates, a second biopsy of the foot lesion (Figure 1), and blood cultures. The patient's condition deteriorated rapidly; cholestasis and septic shock developed, and mechanical ventilation and high doses of vasopressors were required. Fifteen days after resolution of neutropenia and while undergoing antimycotic treatment, the patient died of multiorgan failure caused by systemic fungal infection. Consent was not given for autopsy.

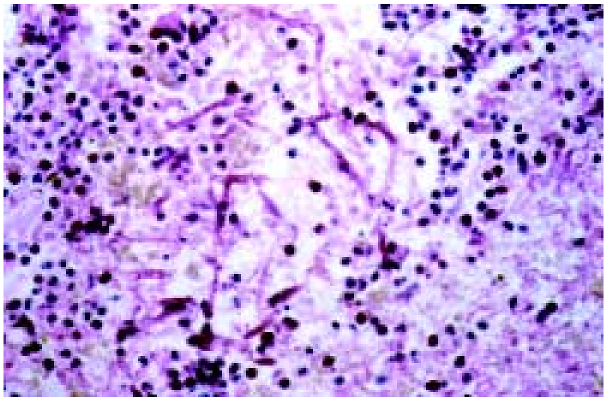

Figure 1. Biopsy of foot lesion: area of extended dermal necrosis with inflammatory infiltration. In the center, PAS-(periodic acid-Schiff stain)positive septate hyphae branching at an acute angle (x400).

Samples were cultivated on Sabouraud agar at 30°C and 37°C in ambient air. Subcultures were prepared on malt yeast agar, oatmeal agar, and potato dextrose agar. The fast-growing, white-to-pale-buff colonies developed at 26°C and 37°C without special requirements.

The micromorphologic features of the mold, cultured 3 to 4 days only, resembled a *Fusarium* or *Acremonium* species (Figure 2a). Supported by subculturing on oatmeal agar with *Lupinus* sp., orange-brown to copper-colored fruiting bodies developed until day 8 (Figure 2b); after 14 days of incubation, these bodies were identified as perithecial ascomata (diameter 200 µm-300 µm). They contained hyaline cylindrical asci, 9 x 80-90 µm, with 8 ascospores each (Figure 2c); the latter were globose to ellipsoidal, pale brown, rough walled with age, and 8-9 x 10 µm. The isolate was subsequently identified at the Centraalbureau voor Schimmelcultures, Baarn, Netherlands, as a small-spored *N. vasinfecta* E.F. Smith var. *vasinfecta* and was deposited under the collection number CBS 101957.

Sequence analysis of the internal transcribed spacer (ITS) region inside the nuclear rDNA was performed. DNA was extracted by sonication in CTAB buffer (cetyl trimethyl ammonium bromide) (2) and amplified with the primer pair ITS5/ITS4. The polymerase chain reaction

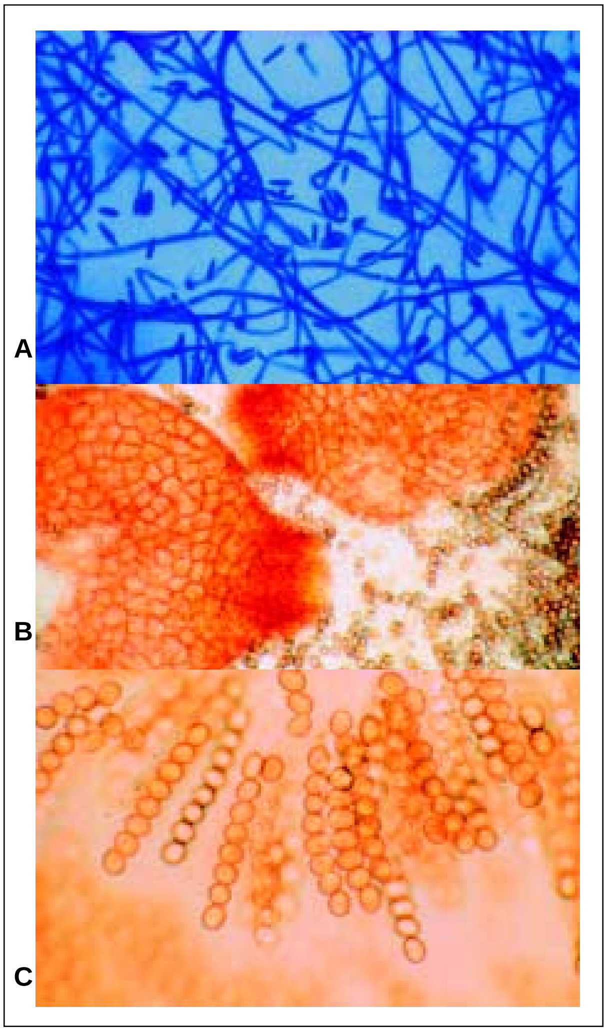

Figure 2. A) *Neocosmospora vasinfecta* after 4 days' subculture on Sabouraud agar (lactophenol cotton blue stain, x400). B) *N. vasinfecta* perithecial ascomata after 4 weeks on oatmeal agar with lupine stem (x160). C) Asci with ascospores of *N. vasinfecta* (x400).

product was purified from agarose gel by using the Gel Extraction Kit (Qiagen GmbH, Hilden, Germany) and directly sequenced with the primers ITS2, ITS3, ITS4, and ITS5 (3). DNA sequencing was performed by the dideoxynucleotide termination method, using the dye deoxy terminator chemistry and the ABI 377 automated sequencer (PE Applied Biosystems, Foster City, CA). The results were compared with the published ITS sequence, GenBank accession no. L36627.

When we compared our results to the published 544-bp ITS sequence, we found differences in three positions: T to C exchange at nucleotide position 72, C to T exchange at position 102, and an insertion of a seventh C between positions 381 and 388.

The MICs of amphotericin B and 5-flucytosine were determined by the ATB Fungus kit (Biomérieux SA, Marcy l'Étoile, France). Susceptibility testing to fluconazole, itraconazole, terbinafine, and voriconazole (Pfizer Inc., New York, NY) was performed by using a casitone-based microdilution system (4). The MIC for itraconazole was simultaneously determined according to the National Committee for Clinical Laboratory Standards (NCCLS) M27-A protocol (5). The inoculum had a final amount of $1x10^4$ conidia per microliter. Incubation time at 35°C was 44 hours for amphotericin B and 5-flucytosine and 30 hours for the other antifungals.

MICs were as follows: amphotericin B > 8 µg/mL$^{-1}$, 5-flucytosine >128 µg/mL$^{-1}$, fluconazole >128 µg/mL$^{-1}$, itraconazole >2 µg/mL$^{-1}$ versus >8 µg/mL$^{-1}$ (according to NCCLS), voriconazole 1 µg/mL$^{-1}$ , and terbinafine 0.125 µg/mL$^{-1}$.

### Conclusions

In the early days of incubation, the micromorphologic features of *N. vasinfecta* isolates may be misleading, suggesting *Fusarium* or *Acremonium* species (1), which could lead to an inadequate therapeutic regimen because of the different amphotericin B susceptibility of these genera. While high-dose amphotericin B intravenously is the first-line therapy in systemic fusarioses, polyene antimycotics are probably not beneficial against infections caused by *N. vasinfecta*, as demonstrated in our patient and in a recently reported immunocompetent patient with posttraumatic *N. vasinfecta* infection (6).

Our patient died of disseminated infection by *N. vasinfecta* despite high-dose antimycotic therapy with amphotericin B and remission of the underlying hematologic malignancy. The in vitro susceptibility test results confirmed the resistance of the isolate against amphotericin B and fluconazole and nearly all of the few other antifungal agents available for systemic therapy. Not enough data on terbinafine are available to allow valid interpretation. In comparison to the MICs of voriconazole against other filamentous fungi such as *Aspergillus fumigatus* (7), the susceptibility for voriconazole in our isolate might not have been sufficient for adequate antifungal treatment.

Neither the mechanism of infection nor the incubation period could be determined. Our patient did not remember any injury, so infection most likely occurred through minimal trauma. Although he might have been infected during his recent stay in Nigeria, infection might have occurred in Africa considerably longer ago: The latency period for *N. vasinfecta* can be several years between infection and the symptomatic stage (8). The infection might never have been detected had the patient remained immunocompetent. Once the infection became systemic, it was likely incurable.

The two previously reported patients (6,8,9), both from Senegal, had been cured by a radical surgical intervention that prevented dissemination of disease, limiting it to the lower extremities. Because of the poor susceptibility of the fungus, complete surgical excision prompted by early diagnosis is the only means to influence the clinical outcome. Currently, no medical treatment option exists for a patient with systemic disease caused by *N. vasinfecta*.

Imported mycoses should be taken into consideration in patients who have traveled or lived abroad, especially former residents of tropical and subtropical countries, even years after their stay abroad. Recent epidemiologic data on plants suggest that persons working in cotton production might also be at risk (10).

This is the first case of disseminated *N. vasinfecta* infection. Physicians may consider this infection as a new differential diagnosis of purulent subcutaneous leg lesions in the severely immunocompromised host.

### Acknowledgments

The authors thank Michael Seibold for performing the antifungal susceptibility tests.

Dr. Cornely is a senior resident and clinical research fellow at the Universitätsklinik Köln, Köln, Germany. His research interests include treatment of hematologic and oncologic malignancies, with a focus on supportive care and infectious diseases in neutropenic hosts.

### References

1. Cannon PF, Hawksworth DL. A revision of the genus Neocosmospora (Hypocreales). Transactions of the British Mycological Society 1984;82:673-88.
2. Wedde M, Müller D, Tintelnot K, De Hoog GS, Stahl U. PCR-based identification of clinically relevant Pseudallescheria/Scedosporium strains. Med Mycol 1998; 36:61-7.
3. White TJ, Bruns T, Lee S, Taylor J. Amplification and direct sequencing of fungal ribosomal RNA genes for phylogenetics. In: Innis N, Gelfand J, White T, editors. PCR protocols: a guide to methods and applications. New York: Academic Press; 1990. p. 315-22.
4. Seibold M, Werner E. Testing susceptibility of Candida species to fluconazole and itraconazole using the microdilution assay. Mycoses 1995;38:443-8.
5. National Committee for Clinical Laboratory Standards (NCCLS). Reference method for broth dilution antifungal susceptibility testing of yeasts. M27-A. Villanova, PA: NCCLS; 1997.
6. Kac G, Piriou P, Gueho E, Roux P, Tremoulet J, Denis M, et al. Osteoarthritis caused by *Neocosmospora vasinfecta*. Med Mycol 1999;37:213-7.
7. Espinel-Ingroff A. In vitro activity of the new triazole voriconazole (UK-109,496) against opportunistic filamentous and dimorphic fungi and common and emerging yeast pathogens. J Clin Microbiol 1998;36:198-202.
8. Ben Hamida F, Achard JM, Westeel PF, Chandenier J, Bouzernidj M, Petit J, et al. Leg granuloma due to *Neocosmospora vasinfecta* in a renal graft recipient. Transplant Proc 1993;25:2292.
9. Chandenier J, Hayette MP, de Bièvre C, Westeel PF, Petit J, Achard JM, et al. Tuméfaction de la jambe à Neocosmospora vasinfecta chez un transplanté rénal. Journal de Mycologie Médicale 1993;3:165-8.
10. Baird R, Carling D. Survival of parasitic and saprophytic fungi on intact senescent cotton roots. Journal of Cotton Science 1998;2:27-34.
